# Acquired Dyslexia in Three Writing Systems: Study of a Portuguese-Japanese Bilingual Aphasic Patient

**DOI:** 10.3233/BEN-2012-119001

**Published:** 2012-03-19

**Authors:** Mirna Lie Hosogi Senaha, Maria Alice de Mattos Pimenta Parente

**Affiliations:** ^1^Member of Behavioral and Cognitive Neurology UnitDepartment of NeurologyUniversity of São Paulo School of MedicineSão PauloBrazil; ^2^Institute of PsychologyDepartment of Developmental and Personality PsychologyFederal University of Rio Grande do SulPorto AlegreBrazil

**Keywords:** Acquired dyslexia, written systems, surface dyslexia, kanji-kana, lexical reading, bilingualism, dissociation

## Abstract

The Japanese language is represented by two different codes: syllabic and logographic while Portuguese employs an alphabetic writing system. Studies on bilingual Portuguese-Japanese individuals with acquired dyslexia therefore allow an investigation of the interaction between reading strategies and characteristics of three different writing codes. The aim of this study was to examine the differential impact of an acquired brain lesion on the reading of the logographic, syllabic and alphabetic writing systems of a bilingual Portuguese-Japanese aphasic patient (PF). Results showed impaired reading in the logographic system and when reading irregularly spelled Portuguese words but no effects on reading regular words and nonwords in syllabic and alphabetic writing systems. These dissociations are interpreted according to a multi-route cognitive model of reading assuming selective damage in the lexical route can result in acquired dyslexia across at least three different writing codes.

## 1. Introduction

The Japanese language uses different writing codes: logographic and syllabic. Studies of premorbidly literate Japanese speakers with acquired dyslexia thus allow an investigation of how script has an effect on the cognitive processes used in the oral reading of different writing systems. In Brazil, the high number of Japanese speakers makes this investigation more intriguing. This is because most Brazilian Japanese speakers also read and write in an alphabetic script – Portuguese – making these speakers tri-scriptal as well as bilingual.

Brazilian Portuguese presents, for the reading, a predominantly alphabetic writing system with few irregularities. This is reflected in the relationship between number of phonemes (26) and letters (23). Most letters represent a univocal relationship between sounds and script that is independent of word context. However, three letters (<x>, <e>, <o>) do present ambiguous relationships, with pronunciation dependent on the intraword context i.e. different sounds depending on the word [27]. For example, the consonant <x> can be read aloud as any one of phonemes /ʃ /, /s/, /ks/ or /z/, as in the examples <faxina>, <máximo>, <sexo> and <exame>. There is also spelling to sound ambiguity in the vowels <e> and <o> when they are in a stressed position in paroxytone words. The vowel <e> can be associated to phonemes /ɛ/ or /e/, as, for example, in <festa> and <vespa>, and the vowel <o> to the phonemes /ɔ/ or /o/, as <cosmo> and <fosco>. Some consonants also have context-dependent relationships. For instance: (1) if the letter <c> is preceded by the vowels <e> or <i>, it is associated with the phoneme

ISSN 0953-4180/12/$27.50 © 2012 – IOS Press and the authors. All rights reserved

/s/, as in the word <Cecília>; (2) if the letter <c> is preceded by other vowels or by a consonant, except the letter <h>, it is converted to the phoneme /k/, as in <cabeça> and <pacto>; and (3) if the letter <c> is preceded by the consonant <h>, both symbols constitute a digraph corresponding to the phoneme /ʃ/.

The Japanese writing system is different to the Portuguese script in several ways. Japanese uses two types of written code: a logographic code that is represented by kanji characters, and a syllabic code, that is represented by the kana script. Kanji characters were originally derived from Chinese ideograms. Kanji characters represent a lexical morpheme of a spoken Japanese word and have phonetic and semantic value (e.g. 車 [kuruma] meaning car). Unlike Chinese characters, kanji has two (or sometimes more) pronunciations: a 'kun' reading, that is the adaptation of the Japanese meaning to Chinese writing and one (or more) 'on' readings, that is the sound transcription from the Chinese language. In the Japanese syllabic system, that is represented by written kana script, each symbol corresponds to a syllable, or to a mora, without meaning (e.g. み [mi], そ [so]). There are two forms of Japanese syllabic writing: hiragana and katakana. Both forms of kana have 46 basic syllables with variations in size of the letter and sign additions leading to 102 syllables. Kana is a completely transparent syllabic writing system. In other words, kana is regular in terms of the correspondences between orthography and phonology.

Although Kanji and kana are different writing systems, they are not alternative systems for communication. It is possible to write a text in Japanese using only syllabic kana. However this happens in the first year of education only. Also, it is not possible to write a meaningful text in Japanese using only kanji. To write a sentence in Japanese, the two writing systems are mixed and engaged simultaneously. In general, open class words – nouns, verbs and adjectives – are written in kanji while the function words – inflections of verbs and adjectives, conjunctions and others – are written in hiragana whereas katakana is used for writing foreign names and foreign words integrated into Japanese.

How can we understand the reading of bilingual, triscriptal speakers? Cognitive neuropsychological studies of oral reading are based on theoretical models that are based on the observation of dissociation between reading and writing tasks as well as variability in the relationship between orthography and phonology [3]. Since the 1970s, studies of patients with acquired dyslexia have revealed dissociations in the reading and writing of regular and irregular words in alphabetic and non-alphabetic writing scripts [42]. Patients may show preservation of irregular word reading with an inability to read low frequency words and nonwords. Other patients can show the opposite pattern i.e. impaired irregular word reading but are able to read nonwords and low frequency regularly spelled words.

Most cognitive models or oral reading assume at least two independent reading pathways: lexical and nonlexical routes. In the lexical route, known words are read as a whole with or without access to the semantic system. In the nonlexical route, segmented reading is used, in which graphemes are converted into their respective phonemes for oral reading. These models assume that whereas oral reading of irregular words is achieved via a lexical reading route, nonwords must be read via a sublexical or a nonlexical pathway. Generally, the lexical route processes familiar words and the nonlexical route processes unfamiliar and infrequent regular words. Such models have been applied across scripts.

One view of the cognitive processes involved in reading holds that processes may vary according to the characteristics of each writing code [18,20,21,42]. However, most studies of acquired dyslexia have investigated (relatively opaque) alphabetic scripts, such as English and French. The spelling irregularities in these two codes may induce lexical reading (e.g. yacht, boeuf). However studies of oral reading in transparent scripts, such as Spanish, Italian, Turkish and Welsh are now increasingly reported [1,2,6,10,11,29–32].

Portuguese is an example of transparent script. Although rare, studies of patients with acquired dyslexia in Portuguese tend to reflect the patterns of impaired lexical and nonlexical reading reported in other alphabetic scripts. Portuguese speaking patients with acquired phonological, deep, surface, semantic and literal dyslexia, are all reported [5,13,25,37–39,41]. The characteristics of these types of acquired dyslexias closely resemble those described in English, French and Spanish readers. Thus, the multi-route model of reading has been used to explain disturbances in Portuguese oral reading.

Less is known about the patterns of acquired dyslexia in patients who speak more than one language (bilingual) or read in more than one type of script (bi-scritpal). Reports of brain damaged patients who present dissociations between reading in logographic and syllabic writing systems have been published [15,22,23, 33–36,40]. Sasanuma (1980) first observed cases of surface dyslexia (impairment to kanji reading with preservation of processing the kana script), phonological dyslexia (impairment in kana nonword reading) and deep dyslexia (disproportionately greater impairment for kana than kanji reading and semantic paralexias in kanji reading) with others subsequently reported [27, 34–36,44].

Caramelli et al. [4] was the first to report a Portuguese-Japanese bilingual aphasic patient who had acquired dyslexia. YI was a premorbidly literate reader in three different writing systems. Japanese was the first acquired language (L1) for YI and Portuguese was the second acquired language (L2). After the brain lesion, YI was able to understand high frequency kanji characters and Portuguese written words if they had a high frequency of occurrence in the script. However, he was unable to understand words written in kana and less familiar low frequency words written in Portuguese. Using the multi-route framework, Caramelli et al. suggested that YI processed high frequency Portuguese words and kanji characters using an intact lexical reading pathway and also argued that YI's inability to read Portuguese nonwords, kana written words and nonwords was a consequence of impairment to the nonlexical reading pathway.

One problem with the interpretation offered by Caramelli et al. is that oral reading of nonwords written in alphabetic and non-alphabetic writing systems may simply be more difficult than reading known words in any language. However, there is evidence of the reverse dissociation in the pattern of oral reading in Portuguese-Japanese speakers i.e. selective impairment of the lexical route with intact nonlexical reading in these patients. In an epidemiological study with Japanese immigrants in Brazil (Nisei), Meguro et al. [20] identified four Portuguese-Japanese bilingual speakers with Alzheimer's disease who showed dissociations between the reading of logographic, syllabic and alphabetical scripts. In these cases, kanji (L2) and Portu- guese (L1) irregular word reading were more affected than both kana and Portuguese regular word and nonword reading. Together with the case report of Caramelli et al. [4], this suggests a "double" dissociation in tri-scriptal Portuguese-Japanese reading and supports the assumption of a multi reading framework available to Portuguese-Japanese speakers. However, due to possible differences in the age of acquisition and premorbid familiarity with alphabetic and non-alphabetic scripts in tri-scriptal speakers, it is not easy to interpret the patterns of dissociation in performance. It is therefore necessary to test the veracity of claims made in prior studies of Portuguese-Japanese speakers with acquired dyslexia.

The aim of the present study was to test the multi-route framework for tri-scriptal oral reading with a Portuguese-Japanese speaker. Specifically, it was of interest to know if the reported dissociation between lexical reading (Kanji and irregular word reading in Portuguese) or nonlexical reading (Kana and nonwords in Portuguese) was selectively impaired following brain damage. A correlation between impaired lexical reading across scripts in a tri-scriptal patient would challenge the hypothesis that the cognitive processes in reading vary according to characteristics of the writing system [18,20,21]. However, if damage to the reading system of a tri-scriptal patient impairs oral reading in one script only, this would be evidence that type of script constrains oral reading in tri-script readers [see 43 for review]. The data from patients who were premorbidly literate in contrasting writing systems: logographic, syllabic and alphabetic such as those reported by Caramelli et al. [4] and Meguro et al. [20] are a strong test of these alternatives [see also 43]. Also, as Nisei patients are bilingual Portuguese and Japanese speakers and also bi-scriptal in one language (Japanese), it is possible to examine if differences in the type of script (syllabic versus logographic) or the status of a script (L1 versus L2) has a greater impact on impaired reading performance. If language status has an effect perhaps because one language is acquired earlier or is more familiar, then we might expect to observe greater impairment to the oral reading of one language with no differences in the performance between syllabic and logographic scripts used in the Japanese language. However, if we observe dissociations in the oral reading performance of a patient between the Japanese scripts, this would be evidence that the type of script has an effect on bi-scriptal reading.

We report here the case of a Portuguese-Japanese bilingual and tri-scriptal patient (PF) with acquired dyslexia. We asked the following questions: (1) does brain damage influence the cognitive mechanisms in oral reading across scripts according to lexical and nonlexical processes independently of the writing system; and (2) in each language, do the patterns of acquired dyslexia observed depend on the unique features of the writing system i.e. is there a dissociation in the Japanese script that is not observed in Portuguese or vice versa? For comparison, tasks were administered to two healthy control subjects, whose language background and sociolinguistic characteristics were very similar to PF.

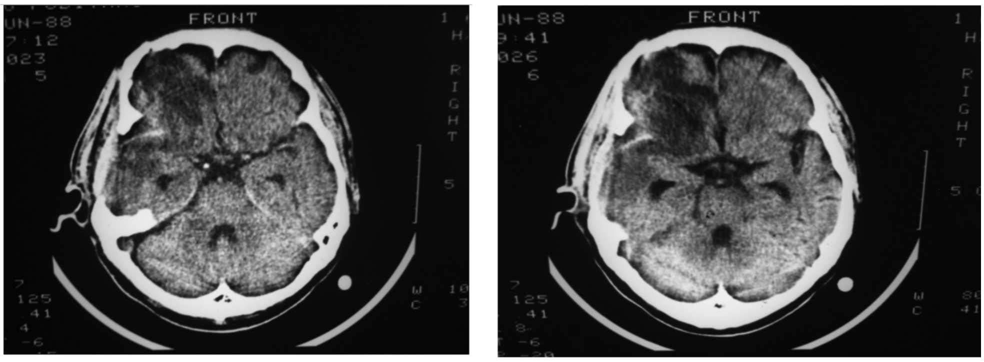

Fig. 1. CT scan of PF showing left frontotemporal lesion after traumatic brain injury.

## 2. Method

### *2.1. Participants*

#### *2.1.1. Case report*

PF is a 48 year-old, right-handed male. At the age of 39, he suffered a traumatic brain injury to the left frontotemporal area (Fig. 1) in a traffic accident, which resulted in Broca's aphasia and right hemiparesis. PF is Brazilian and the son of Japanese parents. His mother tongue was Japanese. During his childhood, teenage years and adult life he used Japanese within the family environment. He acquired the Portuguese language when he started school during early literacy learning. His formal study was completed in the Portuguese language and he graduated with a degree in Electrical Engineering. In the academic environment and subsequently in his work activities, PF used predominately Portuguese for communication. He learned to read and to write Japanese with parent help. He was able to read aloud and write both syllabic and logographic systems of Japanese writing. Prior to his accident, he frequently read magazines and newspapers in Japanese and in Portuguese. Reading Japanese newspapers requires very advanced knowledge of logographic writing. After graduation, PF worked in a Japanese multinational company for 15 years, in which it was necessary to have good Japanese oral and written language proficiency. He made frequent business trips to Japan. He also had good knowledge of a third language, English. English language was not however a focus of this investigation.

#### *2.1.2. Control subjects*

PF's performance on oral reading and other tasks was compared with two healthy controls who have similar sociolinguistic characteristics. Both control subjects were men, right-handed and born in Brazil, and their parents were also Japanese. Their mother tongue was Japanese and their second language was Portuguese acquired when starting school. Their formal study was completed in Portuguese. Both controls were able to read newspapers in Portuguese and Japanese. They presented with no history of oral and written language disturbances or neurological impairment. Control One was a 52 year-old accounting technician (12 years of Japanese education). Control Two was a 53 year-old with an Economics degree who had studied Japanese up until the age of 17 in private schools.

### *2.2. Experimental investigation*

PF and the control subjects were given word and nonword reading aloud tasks (Part I) and specific tasks to examine lexical and nonlexcial oral reading mechanisms (Part II). All evaluations involved written stimuli presented in the Portuguese alphabetic, Japanese syllabic and Japanese logographic writing systems. To examine oral reading, tasks from the Portuguese and Japanese Human Frontier Science Program (HFSP) protocols [14,24] were selected and in addition other specially formulated complementary tasks were also designed to investigate reading disturbance across all three scripts.

*Part I – Reading aloud tasks*

*1. Alphabetic system*

*1.1. Words*

The Portuguese reading aloud task from the HFSP protocol [24] was administered. The test is composed of 180 balanced stimuli to verify the effects of regularity, imageability, frequency, length and class of words. Of 180 stimuli, 40 words have irregular spellings, half of them being composed of irregular words for reading in Portuguese, and the other half corresponding to foreign words used frequently in Brazil, such as <pizza>.

*1.2. Nonwords*

A reading aloud task with 300 nonwords written according to the characteristics of the Portuguese alphabetic system was administered to verify the influence of word length and syllabic structure on oral reading. To examine length effects, nonwords were divided into three stimuli groups (bisyllabic, trisyllabic and polysyllabic) with 100 stimuli each. These groups were then further divided into another five subgroups of 20 stimuli representing the characteristics of the Portuguese writing system: consonant-vowel (CV); consonant-vowel-consonant (CVC); consonant-vowel-vowel (CVV); consonants group and digraph.

*2. Logographic system (kanji)*

*2.1. Words*

The kanji reading aloud task from the Japanese HFSP protocol [14] with 120 words was administered. Only words represented by a single kanji character were selected. Stimuli were chosen according to the linguistic variables of length, imageability, frequency [17], number of strokes (kanji characters up to seven strokes and kanji characters with more than 10 strokes), type (simple and compound) and verbs (conjugated verbs and verbs in the infinitive). Twenty stimuli for each of these variables were selected and, as for the reading aloud task of the Portuguese HFSP protocol [24], stimuli were selected in pairs, modified only by the criteria being tested, while keeping all the others equivalent. Due to the logographic characteristics of kanji and the option of using words represented by only one kanji, it was not possible to create a list of nonwords in the logographic system.

*3. Syllabic system (hiragana and katakana)*

*3.1. Words*

The reading aloud task of real words in hiragana presented 90 stimuli divided into four groups, designed to exam frequency, length, imageability and word class effects. The word reading aloud task in katakana consisted of 60 balanced items, to verify the effects of length, imageability and familiarity. A familiarity criterion was chosen instead of frequency due to the absence of a word frequency norms in katakana at time of testing.

*3.2. Nonwords*

The hiragana nonword reading task comprised 120 stimuli, constituting 40 bisyllabic, 40 trisyllabic and 40 polysyllabic nonwords. In each group, half (20 nonwords) were made up of simple syllables and half, complex syllables. The katakana nonword reading task contained 120 stimuli, testing effects of length and syllabic structure. The number and distribution of stimuli types in this task were equal to the hiragana nonwords reading task.

*Part II – Specific tests – lexical reading mechanisms exam*

*1. Input orthographic lexicon exam*

*1.1. Lexical decision tasks - alphabetic system*

Two lexical decision tasks were used to test the alphabetic system. The first was from the HFSP protocol [24] with 10 items, with two stimuli in each. One stimulus corresponded to an irregular real word and the other, to a nonword with the same syllabic structure and length as the irregular word. Each item was presented separately on a sheet of paper and subjects were requested to indicate the real word. The second lexical decision task was composed of regular words and nonwords presented in a tachistoscopic task. For this task, 39 regular words and 39 nonwords were selected. The nonwords were created in pairs from each real word, taking into consideration the length and the syllabic structure. Each of the 78 stimuli was introduced separately in the center of a computer screen, displayed for 100 milliseconds. The subjects were instructed to hit keys <p> or <o> to indicate if the stimuli represented a real word or a nonword, respectively. In addition, there was an option to hit the letter <i> if they did not know the answer.

*1.2. Lexical decision task – logographic system*

To test the input orthographic lexicon for the logographic system, a similar task to the lexical decision task for the alphabetical code was used. The task consisted of 10 items. Each item had three stimuli, comprising two real kanji characters and a third, false kanji that was derived from components of the other two real kanji characters. The subjects were requested to identify the false kanji.

*1.3. Lexical decision task – syllabic systems*

The lexical decision task from the HFSP protocol [14] in the Japanese syllabic systems had 10 items in hiragana and 10 in katakana. Each item was composed of a real word and a nonword. The subjects were requested to indicate the real word.

*2. Access to semantic system examination*

*2.1. Reading and written comprehension of irregular and foreign words – alphabetic.*

To verify access to the semantic system in Portuguese, a task with 22 Portuguese irregular words and 16 foreign words was devised. The stimuli were presented individually. For each word, reading aloud was requested after which the meaning of the read stimulus was requested. The main objective of this task was to examine access to the semantic system after reading mistakes (regularizations). There are two possibilities: first via the input orthographic lexicon i.e. through the written form of the word; and second, via the output phonological lexicon through the phonological representation of the read word.

*2.2. Reading and written comprehension of kanji – logographic system*

This task was composed of 20 single-character kanji words. The stimuli were presented individually. For each kanji word, reading aloud was requested after which the meaning of the read stimulus was requested. As in the Portuguese version previously described, the objective of this task was to verify access to the semantic system. Due to the transparency of the Japanese syllabic system, tasks to verify access between the input orthographic lexicon and the semantic system with written stimuli in hiragana and katakana were not carried out.

*3. Semantic system examination*

*3.1. Written word comprehension – alphabetic system*

A written word – picture matching task was administered, having frequency of target stimuli as a linguistic variable. A total of 20 words were selected, 10 high-frequency and 10 low-frequency. Participants were requested to identify the picture corresponding to the written word. Among the five picture alternatives, there were: a target picture, a semantic, a visual, a phonological distractor and a picture with no relationship with the target.

*3.2. Written word comprehension – logographic system*

A written word (kanji) – picture matching task was performed. The task contained 20 items and each item was composed of a kanji written word and four picture alternatives: a target, a semantic, a phonological and a visual distractor.

*3.3. Written comprehension – syllabic system*

Two written word – picture matching tasks were performed, one with stimuli in hiragana and another in katakana. Each task contained 20 items and each item had a written word and four picture alternatives, consisting of a picture target and three semantic distractors. The subjects were requested to identify the picture corresponding to the written word.

*4. Output phonological lexicon examination*

*4.1. Homophonic nonword – irregular word matching – Portuguese*

To test the output phonological lexicon in Portuguese, a homophonic nonword – irregular word matching task was applied. This task was composed of 10 items, each containing three stimuli. Among these stimuli, one corresponded to an irregular word; the second to a homophonic nonword to the irregular word; and the third to a nonword visually similar to the irregular word. For instance, one of the items had the following stimuli: <sexo>, <séquisso> and <sejo>. Participants were asked to point the two stimuli with the same phonological representation.

*4.2. Kanji – Katakana matching*

The output phonological lexicon stores the phonological representations of lexical items. Therefore, this memory is not related to the writing code, but to the phonology of the language. Thus, to test the output phonological lexicon in Japanese, a kanji – katakana matching task was administered. In this task, the subjects were requested to match a kanji (Japanese word) to a katakana "nonword" which represented the kanji transcription (the same phonological form of the kanji). Therefore, this second stimulus was a "real oral Japanese word" but a "written nonword" (as previously cited in the Introduction, katakana characters are only used to write foreign words). It was assumed that matching the phonological similarity between the two stimuli would be accomplished by the output phonological lexicon since, (1) kanji is mandatorily processed via the lexical route and (2) a katakana "written nonword" is processed via the nonlexical route. The task had 10 items and each item was composed of a kanji written word and four katakana "nonword" alternatives.

*5. Semantic system and output lexical mechanisms*

*5.1. Picture naming – Portuguese and Japanese*

Picture naming and lexical reading share some lexical-semantic mechanisms. To examine these mechanisms, picture naming tasks were administered in Portuguese and Japanese with 40 pictured objects, balancing the length and familiarity criteria.

*Procedure*

The oral responses were recorded with a tape recorder and the productions transcribed. Performance was analyzed in relation to number and type of errors and compared later with the performance of control participants. Statistical analyses were conducted using Fisher's exact and Chi-square tests to compare proportion of correct responses among the participants. The significance value accepted was $p < 0.05$.

## 3. Results

### 3.1. *Part I – Reading aloud tasks*

#### 3.1.1. *Alphabetic system – words and nonwords*

For word reading aloud in Portuguese, PF produced a relatively high percentage of correct responses (87.8%). He made 22 errors, 18 (81.8%) being a regularization type. Considering the different linguistic variables, PF's performance varied between 90.0% and 100.0% of correct answers across all variables, except for the group of irregular words, of which PF read only 60.0% correctly (see Table 1). Comparison of his performance to controls in irregular word reading showed a significant difference ($x^2 = 14.1$, $p < 0.001$). Sporadic slow reading was observed, chiefly in reading longer words.

On the nonword reading task with alphabetic stimuli, PF read 78.3% correctly. Although performance was lower than controls this was not a significant difference (see Table 1). Among PF's errors, he produced phonemic paralexias most often. Analysis of effects of length and syllabic complexity showed no differences between bisyllabic, trisyllabic and polysyllabic stimuli. Furthermore, there was no effect of syllabic structure (CV, CVC, CVV, digraphs and consonantal groups) on performance.

Table 1
Performance of PF and controls in word and nonword reading aloud tasks in the alphabetic system

| | | % success | |
|---|---|---|---|
| Stimuli | n | PF | Controls |
| Words | | | |
| Regular | 20 | 100.0 | 100.0 |
| Regular (type 2)** | 20 | 90.0 | 100.0 |
| Irregular | 40 | 60.0* | 95.0* |
| High frequency | 10 | 100.0 | 100.0 |
| Low frequency | 10 | 90.0 | 100.0 |
| High imageability | 10 | 100.0 | 100.0 |
| Low Imageability | 10 | 100.0 | 100.0 |
| Short | 10 | 100.0 | 100.0 |
| Long | 10 | 100.0 | 100.0 |
| Verbs in the infinitive | 10 | 100.0 | 100.0 |
| Conjugated verbs | 10 | 100.0 | 100.0 |
| Closed class | 20 | 90.0 | 100.0 |
| Total | 180 | 87.8 | 98.9 |
| Nonwords | | | |
| Total | 300 | 78.3 | 88.5 |

*$p < 0.001$; **regular words for reading, but irregular for writing.

Table 2
Performance of PF and controls on the reading aloud task in logographic and syllabic systems

| | | % success | |
|---|---|---|---|
| Stimuli | n | PF | Controls |
| Words – kanji | | | |
| Total | 120 | 40.0* | 84.2* |
| Words – hiragana | | | |
| Total | 90 | 97.8 | 99.4 |
| Words – katakana | | | |
| Total | 60 | 96.7 | 98.3 |
| Nonwords – hiragana | | | |
| Total | 120 | 87.5 | 87.9 |
| Nonwords – katakana | | | |
| Total | 120 | 74.2 | 82.1 |

*$p < 0.001$.

#### 3.1.2. *Logographic and syllabic system – words and nonwords*

PF's performance was similar to controls in oral reading tasks using stimuli written in syllabic systems: written words and nonwords in hiragana and katakana (see Table 2). However, a significant difference was identified in reading written stimuli in the logographic system: PF read 40.0% of the items correctly while the controls had more than double the number of correct answers compared to PF ($x^2 = 49.9$, $p < 0.001$). Among errors in kanji reading, the majority (81.9%) corresponded to the absence of a response ("I don't know"). Visual mistakes, semantic, formal and phonemic paralexias and unclassifiable mistakes were also observed, albeit in smaller number.

Analyzing PF's performance in kanji reading for each pair of stimuli according to linguistic variables (high frequency versus low frequency; high imageability versus low imageability; number of strokes; length; type and verbs) no effects were found. However, in other analyses considering all stimuli of the protocol (120 kanji words), and comparing the linguistic variables mentioned above, the effect of frequency was significant ($x^2 = 9.5$, $p < 0.05$). PF correctly read 48.9% of high frequency kanji characters and 14.6% of low frequency kanji characters.

In reading aloud of written words in the Japanese syllabic systems, PF correctly read 97.8% of hiragana words and 96.7% of katakana words. Due to the small number of errors, differences in performance according

Table 3
Performance of PF and controls in specific tasks examining lexical reading mechanisms

| | | %success | |
|---|---|---|---|
| Tasks | n | PF | Controls |
| Alphabetic system – Portuguese | | | |
| • Input orthographic lexicon | | | |
| Lexical decision (HFSP) | 10 | 100.0 | 100.0 |
| Lexical decision (taquistoscopic presentation) | 78 | 87.2 | 92.3 |
| • Access to semantic system | | | |
| Comprehension of words read incorrectly | 12 | 100.0 | NA |
| • Semantic system | | | |
| Written comprehension | 20 | 100.0 | 100.0 |
| • Output phonological lexicon | | | |
| Homophonic nonword – irregular word matching | 10 | 90.0 | 100.0 |
| • Semantic system and output lexical mechanisms | | | |
| Picture naming | 40 | 42.5* | 98.8* |
| Logographic system | | | |
| • Input orthographic lexicon | | | |
| Lexical decision (HFSP) | 10 | 80.0 | 100.0 |
| • Access to semantic system | | | |
| Comprehension of kanji characters read incorrectly | 16 | 75.0 | NA |
| • Semantic system | | | |
| Written comprehension | 20 | 85.0 | 100.0 |
| • Output phonological lexicon | | | |
| Homophonic nonword – irregular word matching | 10 | 90.0 | 100.0 |
| Syllabic system | | | |
| • Input orthographic lexicon | | | |
| Lexical decision – hiragana (HFSP) | 10 | 100.0 | 100.0 |
| Lexical decision – katakana (HFSP) | 10 | 100.0 | 100.0 |
| • Semantic system | | | |
| Written comprehension – hiragana | 10 | 100.0 | 100.0 |
| Written comprehension – katakana | 10 | 100.0 | 100.0 |
| Japanese language | | | |
| • Semantic system and output lexical mechanisms | | | |
| Picture naming | 40 | 15.0* | 88.8* |

*$p < 0.001$; NA: not applicable.

to the linguistic variables were not evident. There was no signifiicant difference between PF and controls in hiragana and katakana nonword reading. For hiragana and katakana nonword reading, no significant differences were found in performance in terms of length and syllabic structure.

### 3.2. *Part II – Specific tests – lexical reading mechanisms examination*

#### 3.2.1. *Specific tasks – alphabetic system/Portuguese language*

Comparison between PF and controls on alphabetic writing system tasks revealed no significant differences in lexical decision, written word – picture matching and homophonic nonword – irregular word matching tasks (Table 3). These results suggest that the input orthographic lexicon, the semantic system and the output phonological lexicon – mechanisms that must be involved in reading via the lexical route – were preserved. However, differences between PF and controls were seen in tasks of picture naming and access to the semantic system. PF presented low performance in picture naming, with better results for familiar words in comparison to unfamiliar words.

To evaluate access to the semantic system, a task of reading and written comprehension of irregular and foreign words was applied. The main semantic system from printed words. Testing the written comprehension of words that PF read incorrectly allows verification of how the semantic system is accessed i.e. via input orthographic lexical mechanisms or through output phonological lexical mechanisms. PF read 68.4% words correctly. All of the errors were regularization type. Most striking was the finding that PF provided the correct meaning of all words read incorrectly (see also Wilson and Martinez, this issue). In this task, controls read 98.6% of the stimuli correctly and comprehension of words was preserved.

### 3.2.2. *Specific tasks – logographic and syllabic systems/Japanese language*

To compare PF's performance in the non-alphabetic writing system, specific tasks were designed to investigate lexical reading mechanisms of the logographic and syllabic systems. The main differences between PF and controls were in picture naming and access to the semantic system. In the lexical decision tasks with real and false kanji characters (from the HFSP protocol) PF scored 80.0% correct a score that is considered to be within the normal range [27]. Satisfactory results were also observed on written word comprehension tasks (kanji characters – picture matching) and in the output phonological lexicon tasks (kanji – katakana matching).

On the reading and written comprehension of kanji characters task to verify access to the semantic system, PF read aloud 4 out of the 20 kanji characters appropriately yet was unable to read the other 16 stimuli, giving no response. Despite the absence of response in these 16 kanji characters, PF was able to give the correct meaning of 12, corresponding to 75.0% correct performance. With the four kanji characters for which he did not give the correct meaning, he gave rather vague answers that could be considered close to the target meaning. Therefore, as in the tests of the alphabetic writing system, there was a dissociation between oral reading and written comprehension in the logographic code. Controls read 92.5% kanji characters correctly. PF achieved comparable performance to controls in tasks involving the Japanese syllabic system: lexical decision (input orthographic lexicon) and written word – picture matching task (semantic system) and the kanji – katakana matching task. PF scored 15.0% correct on picture naming in Japanese and correct responses tended to be more familiar objects.

## 4. Discussion

### 4.1. *Portuguese writing system: acquired dyslexia with impairment in the lexical route*

PF had difficulty reading irregular words, producing mainly regularization errors. These data suggest an impairment to the lexical route and preserved use of the nonlexical reading route when reading in Portuguese. His hesitation, slow reading with longer words and performance in nonword reading were also suggestive of reading via the nonlexical pathway. Results from PF's nonword reading task may appear to contradict this claim. However, PF and controls achieved similar levels of performance in nonword reading (see Manarini [19] who reported performance from 87.0% to 95.5% correct in nonword reading by undergraduates). Lower than expected performance in nonword reading may be due to stimuli complexity, and unfamiliarity with the task. PF's performance on tests of irregular word reading was significantly worse than controls. Thus, we contend PF is more impaired when reading via the lexical pathway than the nonlexical pathway.

Considering all words in the reading aloud task of the alphabetical system, PF's performance was relatively high for reading. This may be a consequence of the majority of words containing a regular correspondence between orthography and phonology in Portuguese. Coltheart et al. [7] proposed a list of nine symptoms in surface dyslexia based on anglophone readers. Of all characteristics, the authors considered better regular word than irregular word reading to be a necessary and sufficient symptom for a diagnosis of surface dyslexia. According to these criteria, PF presented the necessary and sufficient symptoms for diagnosis of surface dyslexia in Portuguese. A further striking feature of performance was that reading aloud of irregular words was accompanied by intact comprehension of the same words. It is therefore possible to claim that in Portuguese (as in English and other alphabetic writing systems), surface dyslexia can be observed without damage to the semantic system. We note here that the opposite pattern – damage to the semantic system without surface dyslexia in Portuguese – has also been reported [37,38] as in other languages (see also Wilson et al.).

### 4.2. *Japanese: reading impairment in the logographic and preservation of syllabic systems*

PF displayed a reading impairment in the logographic writing system – however, performance with the syllabic script is no different to controls. This clear dissociation in reading aloud within the Japanese writing system raises some issues. The first is that even though oral reading within a language using two different scripts may be dissociable as shown in previous studies of Japanese patients, it is not clear whether this is evidence of an effect of type of script, an effect of task difficulty or of a selective impairment to one pathway and not to the other. Given several studies have shown that bilingual speakers do not necessarily manifest the same language disorders with the same severity in both of their languages (non-parallel language recovery), the dissociation found in PF's reading in Japanese might shown that each written system is represented (organized) in different neural networks in the brain. However, differences in the patterns of recovery in bilingual aphasia may reflect the language status (L1 versus L2), age of acquisition, familiarity, or dominance. Although these confounding effects may be reduced for bi-script readers who speak a common language such as PF, there is still a problem when interpreting data from biscript patients as these cases may show patterns of recovery that reflect language status. We contend that impairment to the oral reading of kanji likely reflects damage to a lexical reading process and does not reflect impaired reading of a non-lexical process. However, we do not claim dissociations in functional reading routes in Japanese necessarily reflect independent reading systems for kanji and kana at the neural level.

A different issue is whether or not brain damage influences common oral reading processes independently of the writing code presented for testing. Thus, characteristics of PF's acquired dyslexia in the Japanese script – selective impairment in kanji reading and preserved performance in reading of words and nonwords written in the Japanese syllabic transparent code – can be interpreted as a better preserved nonlexical pathway compared to the lexical pathway. Given the same interpretation was offered for PF's oral reading impairments in written Portuguese, it seems redundant to distinguish differences in script to explain PF's impaired reading performance. In other words, if his performance reflects selective damage to the lexical pathway in a common oral reading network, this accounts for the patterns of reading dissociation across scripts. Thus, the co-incidence of impaired lexical reading (Kanji and irregular word reading in Portuguese) and preserved nonlexical reading (Kana and nonwords in Portuguese) suggests no effect of script on PF's reading.

### 4.3. *Analyses of acquired dyslexia in the three writing systems*

The claim that damage to the reading system influences cognitive processes across writing systems is supported by the analysis of PF's impaired and preserved performance in reading and writing: impairment of the lexical route is responsible for low performance in reading kanji and Portuguese irregular words. On the other hand, performance when reading words in the transparent syllabic system and regular words and nonwords of the alphabetic Portuguese system shows nonlexical reading is intact. Together with patients reported by Meguro et al. [20] and Caramelli et al. [4], the present data support the claim that Portuguese-Japanese speakers enjoy multiple cognitive mechanisms for oral reading.

Analysis of PF's acquired dyslexia in the three writing systems is compatible with the view that type of script has little impact on biscriptal reading and does not sustain the view that different writing codes of a biscriptal reader have impact on impaired reading performance. Instead it seems that reading mechanisms (both lexical and nonlexical) are shared for the two different scripts. Damage to the oral reading system for PF resulted in more impairment to shared lexical reading mechanisms than to shared nonlexical reading mechanisms thus explaining problems reading irregularly spelled words in Portuguese and Japanese kanji as well as preserved ability to read kana and alphabetic nonwords.

The reverse analysis can be made of the case YI reported by Caramelli et al. [4] who had good performance in written comprehension of the logographic system and high frequency words in the Portuguese writing system, but was unable to process words and nonwords in the syllabic system and nonwords alphabetic system. Caramelli et al. [4] interpreted YI's data according to a multi route reading model proposed for reading in English. According to them, YI was capable of processing kanji and frequent words of Portuguese lexically, but was unable to process stimuli through print-sound conversion. We would interpret PF's data in the opposite way i.e. PF was not capable of processing kanji and irregularly spelled words of Portuguese lexically, but he was able to process kana and nonwords via print-sound conversion. Therefore manifestations of YI's and PF's acquired dyslexias (both tri-scriptal readers) supports a double dissociation in Portuguese - Japanese oral reading. YI presented better lexical reading than nonlexical reading while PF presented the opposite pattern of reading impairment. Crucially the same pattern of performance was observed across written codes for both cases, with no effect of script.

No regularization errors were observed in PF's Japanese reading. Legitimate Alternative Reading of Components (LARC) errors are a type of regularization error and are observed in patients with acquired surface dyslexia in Japanese [12,26]. These errors correspond to incorrect selection of phonological representations of one or more character components. When reading kanji, LARC errors correspond to the selection of possible pronunciations (on/kun readings). Determining which pronunciation must be used will depend on the context that a kanji is used in two-character kanji words. LARC errors could not observed here because only single-character kanji words are tested with the HFSP protocol [14].[1] And in this case, all the possible kanji pronunciations can be accepted as correct.

It is important to note that if we were to consider PF's performance on the reading of syllabic system only, we might conclude that PF did not have any disturbances in the reading of syllabic code. Hiragana and katakana word reading aloud was satisfactory and few errors were found. Also, performance when reading nonwords written in a syllabic system was similar to controls. Drawing on these data, it may be possible to follow Ardila's [1] and Karanth's [16] arguments that reading in a script with a transparent code requires only a nonlexical route. However, Ruiz et al. [32] reported two cases of Spanish readers with deep dyslexia. This conflicts with Ardila's [1] position that Spanish readers would not present deep or surface dyslexia. Other hispanophonic case reports confirm the possibility that readers of a transparent orthographic code seem to read via a lexical pathway [9–11].

The transparent nature of the script, in either syllabic or alphabetic scripts, does not seem to mandate that readers will only read using a nonlexical pathway. Many studies [6,10,11,32] show evidence of lexical reading in transparent orthographies suggesting multi-route reading models are appropriate for interpreting patterns of acquired reading impairments. Chiacchio et al. [6] reported acquired reading impairments in an Italian speaker [see also other papers in this Special Issue]. They described a case of primary progressive aphasia with surface dyslexia. Instead of regularization errors when reading irregular words, the authors found accent mistakes and proposed this is an aspect of Italian word reading that cannot be based on grapheme-phoneme conversion rules.

The high number of correct responses in PF's reading of words written in the syllabic system (136/140) as well as by Sasanuma's [35] patient, SU, (169/170), with surface dyslexia, may appear as absence of reading disturbance in the syllabic system. A lexical route dysfunction does not cause much impact in transparent code readers, since it is possible to read transparent writing codes appropriately through a nonlexical pathway, but this does not necessarily mean that there is no failure in the lexical route or that transparent code readers can only read via a single route.

Dysfunction in patterns of lexical reading in patients who read with a transparent script is different to readers of deep orthographies. Readers of transparent scripts might not produce classic regularization errors and can read words and nonwords appropriately. But mistakes in accent and hence stress and slow reading speed are additional symptoms. A dysfunction in the semantic system impairs written word comprehension. Therefore, other abilities besides reading aloud should be investigated to examine the presence of failures in the lexical route as demonstrated in the study of Ferreres et al. [10].

### 4.4. Characterization of lexical reading dysfunction

In the experimental test – Part II of this study specific tasks were performed to evaluate cognitive mechanisms involved in PF's lexical reading. The investigation showed that the cognitive dysfunction is localized on the output lexical mechanisms of the lexical route, more specifically in access to the output phonological lexicon from the semantic system and from the input orthographic lexicon for alphabetic, syllabic and logographic codes. The investigations demonstrate intact input lexical mechanisms to the semantic system. PF had satisfactory performance in lexical decision (which investigates the input orthographic lexicon) and written word comprehension (which investigates the semantic system). In addition, data from tasks examining access to the semantic system corroborat the hypothesis of the integrity of the input lexical mechanisms to the semantic system. In the alphabetic system, concerning the task to verify access to the semantic system, PF was able to define irregular and foreign word meanings for words that he had read aloud incorrectly. This behavior suggests that the patient read irregular words using the nonlexical route, but accessed word meaning via the input orthographic lexicon and the semantic system. This interpretation can be maintained, as if the patient accessed the semantic system through output lexical mechanisms, he would access the word meaning by the phonological representation of the irregular word read incorrectly. For instance, PF read the foreign word <pizza> incorrectly. He performed regularization and evoked aloud the word [piza], however he explained the meaning of the word <pizza> correctly. In this example, if PF had accessed the semantic system by the output phonological lexicon, he would state that the

[1]Sasanuma [34,35] described patients MU and SU with impairment in lexical reading. These patients produced semantic errors in kanji reading.

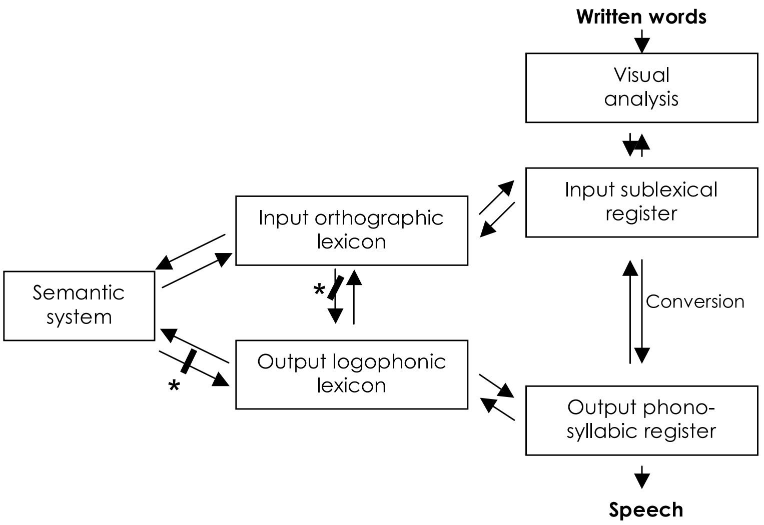

Fig. 2. Dual-route cognitive model of reading showing PF's functional disturbances (*) in access to the output logophonic lexicon from the semantic system and from the input orthographic lexicon (adapted and complemented from Rapcsak et al. [31].)

word [piza] refers to the verb "step" (<pisa>/[piza] in Portuguese) or to the leaning tower in Italy.

On the same task in the logographic system, PF was able to provide meaning for incorrectly read words showing preserved written comprehension of kanji characters. The data suggest the input lexical processes to the semantic system are relatively preserved, and the deficits in lexical reading in both alphabetic and logographic systems are due to impairment in post-semantic lexical processing.

Post-semantic lexical processing involves phonological representations of stimuli. Therefore post-semantic lexical processes do not depend on the orthographic, logographic or syllabic or alphabetic nature of stimuli. Thus, tasks that test the phonological lexicon and the output lexical mechanisms suit the oral language, independently of written code. Regarding output lexical mechanisms, PF performed well on tasks to verify the output phonological lexicon in Portuguese (homophonic nonword – irregular word matching) and in Japanese (kanji – katakana matching). The output lexical mechanisms were also tested through picture naming tasks. PF's low performance in Japanese and Portuguese in these tasks can neither be attributed to failures in perceptual mechanisms nor to the semantic system, given the absence of failures in visual picture recognition along with preservation of the semantic system examined in the written comprehension tasks. Therefore, we propose the hypothesis that PF's naming impairment is correlated with damage to the lexical reading route and is post-semantic and prior to the phonological lexicon. The characteristics of PF's spontaneous speech in Portuguese and in Japanese (non-fluent) and the type of mistakes in some of the formal tests (absence of responses in kanji reading and Portuguese and Japanese picture naming) point to a locus in lexical reading due to dysfunction in access to the phonological output lexicon from the semantic and the orthographic input lexicon (see Fig. 2).

To support this claim we compared the results from PF in some tasks with those obtained by the patient SU, described by Sasanuma [35] who presented surface dyslexia in Japanese (Table 4). Patient SU had Wernicke's aphasia, fluent speech and preserved word comprehension. Similar to PF, SU presented difficulties in the picture naming tasks and in kanji reading. However, after a qualitative analysis of errors in the tasks, it seems errors differed between SU and PF. Whereas PF presented a high number of no responses in picture naming and kanji reading, SU showed a high incidence of circumlocutions in naming and irrelevant errors (substitution of the target word by another word with no semantic or formal relationship to the target), circumlocutions and semantic paralexias in kanji reading. SU presented only 2.9% of no responses in kanji reading while in picture naming, irrelevant errors together with the absence of response comprised 19.6% errors.

Sasanuma [35] claimed SU had impairment in the output phonological lexicon. Errors showed the patient had no difficulty in accessing the phonological representations stored in the output phonological lexicon. However, a functional disturbance in the output phonological lexicon itself, explained failures including circumlocutions and semantic mistakes. The

Table 4
Data comparison for PF and SU (described by Sasanuma [35]) in linguistic abilities

| | PF (Portuguese data) | PF (Japanese data) | SU (Japanese data) |
|---|---|---|---|
| Aphasia type | Broca | Broca | Wernicke |
| Fluency | Non-fluent | Non-fluent | Fluent |
| Word oral comprehension (number and percentage correct) | 9/9 (100.0%) | 8/9 (88.9%) | 20/20 (100.0%) |
| Picture naming (number and percentage correct) | 17/40 (42.5%) | 6/40 (15.0%) | 49/100 (49.0%) |
| Types of erros in naming task | Absence of response – 21/23<br>Semantic paraphasia – 1/23<br>Verbal paraphasia – 1/23 | Absence of response – 33/34<br>Categorization – 1/34 | Circumlocution – 31/51<br>Semantic paraphasia – 7/51<br>Irrelevant/Absence – 10/51<br>Phonemic paraphasia – 2/51 |
| Kanji reading | – | 48/120 (40.0%) | 26/130 (20.0%) |
| Types of errors in kanji reading | – | Absence of response – 57/72<br>Unclassifiable – 5/72<br>Visual – 5/72<br>Semantic paralexia – 2/72<br>Formal paralexia – 1/72<br>Phonemic paralexia – 1/72 | Irrelevant – 47/104<br>Circumlocution – 22/104<br>Semantic paralexia – 15/104<br>Neologism – 14/104<br>On/Kun – 3/104<br>Absence of response – 3/104 |

comparison between SU and PF corroborates our hypothesis that PF's functional disorder is localized in access to the output phonological lexicon. As PF had an access dysfunction, he did not retrieve phonological representations stored in the output phonological lexicon, presenting as poor kanji reading and picture naming, and absent responses. In contrast, SU presented with preserved access between the semantic system and the output phonological lexicon, therefore SU was able to retrieve the phonological representations of lexical items stored in the output phonological lexicon. However, as SU presented a cognitive dysfunction in the output phonological lexicon, she produced inadequate oral answers in kanji reading and picture naming.

The present case study allowed us to test the multi route reading model to explain disturbance of a bilingual patient tri-scriptal reader across three different writing systems. Impairment in lexical reading was verified in the reading the written codes in Portuguese and Japanese. Critically in both languages reading impairment was impaired in a similar way. Moreover, there was evidence that the same functional disturbance in access to the phonological output lexicon from the semantic system and from the orthographic input lexicon was responsible for the acquired dyslexia in three writing systems. Some multiple route reading models assume a third non-semantic lexical pathway available to support the oral reading of irregularly spelled words and logographic characters without access to the meaning of those characters. PF was able to read via meaning albeit at an impaired level. To test the veracity of the lexical non-semantic pathway in biscriptal reading in a Japanese-Portuguese speaker it will be necessary to find a patient who can read aloud words without access to meaning in both languages. No such case has yet been reported.

In sum, the impact of reading disturbance in bilingual speakers who are tri-scriptal readers varies according to the division of labour between the use of the lexical reading and non-lexical reading pathways. We found evidence of a correlated reading impairment to kanji reading and written words that are irregularly spelled in Portuguese in addition to an impairment to access to phonological output in both languages. We locate the damage to impairment in a common lexical reading pathway. There was no evidence of an effect of language status (L1 versus L2) or an effect of type of script on PF's impaired reading performance (for a similar conclusion see Tainturier et al. and Druks et al. this volume).

## Acknowledgments

We are grateful to PF and his wife for their time and collaboration.

## Appendix

### List of kanji stimuli in the reading aloud task

| N. | Kanji | Meaning | On-reading | Kun-reading | PF's response | Type of response |
|---|---|---|---|---|---|---|
| 1 | 虫 | insect | chū | mushi | mushi | correct |
| 2 | 体 | body | tai, tei | karada | tai | correct |
| 3 | 火 | fire | ka | hi, ho | hi | correct |
| 4 | 金 | gold | kin, kon | kane, kana | kin | correct |
| 5 | 頭 | head | tō, zu, to | atama, kashira | atama | correct |
| 6 | 書く | write | sho | ka-ku | kaku | correct |
| 7 | 干す | dry | kan | ho-su, hi-ru | I don't know | absence of response |
| 8 | 時 | time | ji | toki | ji | correct |
| 9 | 麦 | wheat | baku | mugi | I don't know | absence of response |
| 10 | 市 | city | shi | ichi | ichi | correct |
| 11 | 表 | express | hyō | omote, arawa-su, arawa-reru | I don't know | absence of response |
| 12 | 様 | formal title | yō | sama | sama | correct |
| 13 | 男 | man | dan, nan | otoko | otoko | correct |
| 14 | 事 | affair | ji, zu | koto | I don't know | absence of response |
| 15 | 港 | harbor | kō | minato | I don't know | absence of response |
| 16 | 原 | field | gen | hara | I don't know | absence of response |
| 17 | 株 | stock | | kabu | I don't know | absence of response |
| 18 | 指 | finger | shi | yubi, sa-su | yubi | correct |
| 19 | 母 | mother | bo | haha | haha | correct |
| 20 | 届く | deliver | | todo-keru, todo-ku | I don't know | absence of response |
| 21 | 米 | rice | bei, mai | kome | I don't know | absence of response |
| 22 | 氷 | ice | hyō | kōri, hi | I don't know | absence of response |
| 23 | 舌 | tongue | zetsu | shita | I don't know | absence of response |
| 24 | 年 | year | nen | toshi | nen | correct |
| 25 | 題 | title | dai | | dai | correct |
| 26 | 球 | sphere | kyū | tama | kyu | correct |
| 27 | 囲う | surround | i | kako-mu, kako-u | I don't know | absence of response |
| 28 | 毛 | hair | mō | ke | ke | correct |
| 29 | 女 | woman | jo, nyo, nyō | onna, me | onna | correct |
| 30 | 紙 | paper | shi | kami | kami | correct |
| 31 | 地 | ground | chi, ji | | chi | correct |
| 32 | 風 | wind | fū, fu | kaze, kaza | kami | verbal paralexia |
| 33 | 組 | association | so | ku-mu, kumi | I don't know | absence of response |
| 34 | 馬 | horse | ba | uma, ma | uma | correct |
| 35 | 貝 | shellfish | | kai | I don't know | absence of response |
| 36 | 黒 | black | koku | kuro, kuro-i | I don't know | absence of response |
| 37 | 岩 | boulder | gan | iwa | yama | semantic/visual |
| 38 | 池 | pond | chi | ike | ike | correct |

| | | | | | | |
|---|---|---|---|---|---|---|
| 39 | 鼻 | nose | bi | hana | I don't know | absence of response |
| 40 | 岸 | beach Buddhist | gan | kishi | I don't know | absence of response |
| 41 | 寺 | temple | ji | tera | toki | visual |
| 42 | 回したい | times | kai, e | mawa-ru, mawa-su | I don't know | absence of response |
| 43 | 子 | child | shi, su | ko | ko | correct |
| 44 | 玉 | ball | gyoku | tama | kyu | correct |
| 45 | 身 | body | shin | mi | I don't know | absence of response |
| 46 | 波 | wave | ha | nami | I don't know | absence of response |
| 47 | 板 | board | han, ban | ita | I don't know | absence of response |
| 48 | 吹く | blow | sui | fu-ku | kiku | verbal/semantic (?) |
| 49 | 夜 | night | ya | yo, yoru | yoru | correct |
| 50 | 根 | root | kon | ne | me | verbal paralexia |
| 51 | 門 | gate | mon | kado | mom | correct |
| 52 | 葉 | leaf | yō | ha | I don't know | absence of response |
| 53 | 光 | ray | kō | hika-ru, hikari | I don't know | absence of response |
| 54 | 島 | island | tō | shima | shima | correct |
| 55 | 管 | pipe | kan | kuda | I don't know | absence of response |
| 56 | 歯 | tooth | shi | ha | I don't know | absence of response |
| 57 | 野 | field | ya | no | I don't know | absence of response |
| 58 | 魚 | fish | gyo | uo, sakana | I don't know | absence of response |
| 59 | 谷 | valley | koku | tani | I don't know | absence of response |
| 60 | 本 | book | hon | moto | I don't know | absence of response |
| 61 | 村 | village | son | mura | mura | correct |
| 62 | 空 | sky | kū | sora, a-ku, a-keru, kara | sora | correct |
| 63 | 店 | shop | ten | mise | I don't know | absence of response |
| 64 | 戸 | door | ko | to | to | correct |
| 65 | 疲れる | exhausted | hi | tsuka-reru, tsuka-rasu | I don't know | absence of response |
| 66 | 道 | road | dō, tō | michi | michi | correct |
| 67 | 竹 | bamboo | chiku | take | mura | visual |
| 68 | 作らせる | make | saku, sa | tsuku-ru | shiraseru | incorrect |
| 69 | 刺す | thrust | shi | sa-su, sa-saru | osu | verbal paralexia |
| 70 | 炎 | flame | en | honō | I don't know | absence of response |
| 71 | 心 | mind | shin | kokoro | kokoro | correct |
| 72 | 足 | leg | soku | ashi, ta-riru, ta-ru, ta-su | ashi | correct |
| 73 | 橋 | bridge | kyō | hashi | I don't know | absence of response |
| 74 | 都 | capital | to, tsu | miyako | I don't know | absence of response |
| 75 | 富 | wealth | fu, fū | to-mu, tomi | I don't know | absence of response |
| 76 | 船 | boat | sen | fune, funa | I don't know | absence of response |
| 77 | 家 | house | ka, ke | ie, ya | I don't know | absence of response |
| 78 | 公 | Public | kō | ōyake | ko | correct |
| 79 | 所 | Place | sho | tokoro | tokoro | correct |

| | | | | | | |
|---|---|---|---|---|---|---|
| 80 | 花 | Flower | ka | hana | I don't know | absence of response |
| 81 | 炭 | charcoal | tan | sumi | I don't know | absence of response |
| 82 | 角 | horn | kaku | kado, tsuno | I don't know | absence of response |
| 83 | 糸 | thread | shi | ito | I don't know | absence of response |
| 84 | 位 | rank | i | kurai | tai | incorrect |
| 85 | 皮 | skin | hi | kawa | hi | correct |
| 86 | 血 | blood | ketsu | chi | I don't know | absence of response |
| 87 | 油 | oil | yu | abura | I don't know | absence of response |
| 88 | 顔 | face | gan | kao | kao | correct |
| 89 | 書きましょう | write | sho | ka-ku | kakimashou | correct |
| 90 | 教えている | teach | kyō | oshi-eru, oso-waru | oboiteiru | semantic |
| 91 | 薬 | medicine | yaku | kusuri | I don't know | absence of response |
| 92 | 売っている | sell | bai | u-ru, u-reru | I don't know | absence of response |
| 93 | 森 | florest | shin | mori | mori | correct |
| 94 | 砂 | sand | sa, sha | suna | I don't know | absence of response |
| 95 | 盗む | steal | tō | nusu-mu | I don't know | absence of response |
| 96 | 首 | neck | shu | kubi | kao | semantic |
| 97 | 夫 | husband | fu, fū | otto | I don't know | absence of response |
| 98 | 元 | origin | gen, gan | moto | moto | correct |
| 99 | 充てる | fill | jū | a-teru | shiteru | incorrect |
| 100 | 豆 | beans | tō, zu | mame | I don't know | absence of response |
| 101 | 林 | woods | rin | hayashi | I don't know | absence of response |
| 102 | 土 | soil | do, to | tsuchi | to | correct |
| 103 | 水 | water | sui | mizu | mizu | correct |
| 104 | 実 | truth | jitsu | mi, mino-ru | I don't know | absence of response |
| 105 | 毛 | hair | mō | ke | I don't know | absence of response |
| 106 | 行くだろう | go | kō, gyō, an | i-ku, yu-ku, okona-u | ikudarou | correct |
| 107 | 会うでしょう | find | kai, e | a-u | audeshou | correct |
| 108 | 鳥 | bird | chō | tori | tori | correct |
| 109 | 車 | car | sha | kuruma | kuruma | correct |
| 110 | 海 | sea | kai | umi | I don't know | absence of response |
| 111 | 雲 | cloud | un | kumo | I don't know | absence of response |
| 112 | 出ます | exit | shutsu, sui | de-ru, da-su | demasu | correct |
| 113 | 木 | tree | boku, moku | ki, ko | I don't know | absence of response |
| 114 | 話 | talk | wa | hana-su, hanashi | hanashi | correct |
| 115 | 柱 | pillar | chū | hashira | I don't know | absence of response |
| 116 | 西 | west | sei, sai | nishi | I don't know | absence of response |
| 117 | 立ちます | stand up | ritsu, ryū | ta-tsu, ta-teru | machimasu | verbal |
| 118 | 耳 | ear | ji | mimi | I don't know | absence of response |
| 119 | 敬する | offer | kei | uyama-u | I don't know | absence of response |
| 120 | 見ます | see | ken | mi-ru, mi-eru, mi-seru | mimasu | correct |
